# The effect of mildly stimulated cycle versus artificial cycle on pregnancy outcomes in overweight/obese women with PCOS prior to frozen embryo transfer: a retrospective cohort study

**DOI:** 10.1186/s12884-022-04728-6

**Published:** 2022-05-07

**Authors:** Lu Guan, Haicui Wu, Chaofeng Wei, Conghui Pang, Danqi Liu, Xiaona Yu, Shan Xiang, Fang Lian

**Affiliations:** 1 Shandong University of Traditional Chinese Medicine, Jinan, China; 2 Affiliated Hospital of Shandong University of Traditional Chinese Medicine, Jinan, China

**Keywords:** Polycystic ovary syndrome, Frozen embryo transfer, Endometrial preparation, Artificial cycle, Mildly stimulated cycle, Overweight, Obesity

## Abstract

**Background:**

Previous studies have shown that frozen embryo transfer (FET) resulted in increased live birth rates (LBR) and reduced the risk of ovarian hyperstimulation syndrome (OHSS) than did fresh embryo transfer in women with polycystic ovary syndrome (PCOS). In addition, overweight/obese women with PCOS are at increased risk of subfertility and complications of pregnancy, compared with normal-weight women. The ovarian stimulation and artificial hormone regimes are the two more commonly used endometrial preparation protocols in PCOS patients.This retrospective study aims to compare the pregnancy outcomes of mildly stimulated cycles (mSTC) and artificial cycles (AC) prior to FET in overweight/obese women with PCOS.

**Methods:**

A retrospective analysis was conducted in overweight/obese women with PCOS who underwent their first FET cycles from January 2018 to December 2020. Two endometrial preparation protocols were used: the mildly stimulated cycles (*N* = 173) and the artificial cycles (*N* = 507). All pregnancy outcomes were analyzed by Student’s *t*-test, Chi-square (*χ*^*2*^) statistics and multivariable logistic regression analyses.

**Results:**

This study enrolled 680 cases of FET cycles. The mSTC group exhibited significantly higher LBR compared with the AC group (49.7% vs. 41.0%; *P* = *0.046*), while the rate of miscarriage was significantly lower (6.4% vs. 23.0%; *P* < *0.001*). No statistically significant differences were observed in positive pregnancy rate (57.8% vs. 60.0%, *P* = *0.618*), clinical pregnancy rate (54.3% vs. 55.6%, *P* = *0.769*), and ectopic pregnancy rate (2.1% vs. 3.2%, *P* = *0.860*) between two groups. After adjusting for possible confounding factors, multivariate logistic regression analysis also yielded similar results.

**Conclusions:**

For overweight/obese women with PCOS, mSTC-FET demonstrated a higher LBR and a lower pregnancy loss rate than that in the AC-FET. When considering the most cost-effective treatment with the least adverse effects on patients, the mSTC for FET endometrial preparation may be considered. To corroborate our findings, additional prospective randomized clinical trials with larger sample sizes are required.

**Supplementary Information:**

The online version contains supplementary material available at 10.1186/s12884-022-04728-6.

## Background

Polycystic ovary syndrome (PCOS) is the most common endocrine and metabolic disease in women of reproductive age, with an incidence as high as 10% [1]. PCOS is often concomitant with obesity, oligo-or amenorrhea, hormonal abnormality, and infertility. Obesity is closely associated with PCOS and is critical in PCOS development and progression. A study from Spain revealed that PCOS might be more prevalent among overweight and obese women [2], but its incidence rate varied substantially across regions and ethnic groups [3]. In addition, several studies have indicated that PCOS disease status significantly increases the risk of obesity [4–6]. Menstrual disturbances were present in half of obese women with PCOS [4]. Both overweight and obesity significantly reduced the pregnancy rate [4] as well as increased the risk of miscarriage and preterm birth in PCOS patients [7]. In addition, they were associated with a high rate of pregnancy complications such as gestational diabetes mellitus and gestational hypertension [8].

PCOS is the most common cause of anovulatory infertility, accounting for approximately 80% [9]. When traditional treatment methods fail to produce satisfactory results, assisted reproductive techniques (ART) are considered final treatments for PCOS patients, including controlled ovarian hyperstimulation (COH) and embryo transfer. With the refinement of vitrification techniques and ongoing adoption of single embryo transfer strategies, the number of frozen embryo transfer cycles has drastically increased [10]. Due to the increased use of ovulation induction drugs on PCOS, the risk of ovarian hyperstimulation syndrome (OHSS) in these patients increases. In addition to personalized ovulation induction protocols and single embryo transfer, a freeze-all policy is the better choice for PCOS patients seeking to reduce OHSS risk [11]. Previous studies have indicated that this freeze-all strategy significant increases pregnancy frequency rates and live birth rates (LBR) while also significantly reducing the risk of OHSS, which can occur in patients undergoing in vitro fertilization (IVF) and ovarian stimulation, potentially leading to death [12, 13]. FET outcomes depend on many factors, including embryonic quality, endometrial receptivity, and their synchronization. Compared with fresh embryo transfer, FET is closer to the hormone environment of natural pregnancy [13], avoiding the adverse effects of controlled ovarian hyperstimulation on embryo-endometrium synchronization [14].

Different endometrial preparation regimes have been applied to increase endometrial receptivity in FET cycles, including natural, stimulated (STC), and artificial cycles (AC). In the natural cycle, endometrial preparation depends on endogenous steroid hormones produced by developing follicles, similar to natural physiological state. The optimal embryo transfer stage can be determined by monitoring the peak of endogenous luteinizing hormone or ovulation time. Endometrial preparation using a natural cycle may appear more acceptable and cost-effective due to the absence of injections. However, this method is only appropriate for patients with regular menstruation. It might be less convenient for PCOS patients due to the required early medication intervention to maintain regular menstrual periods and more monitoring to determine the appropriate day for embryo transfer. It might also be less convenient for centers due to reduced flexibility [15, 16]. Therefore, the ovarian stimulation protocol and artificial hormonal endometrial preparation are the two most often used endometrial preparation regimens for PCOS patients. A meta-analysis including different regions and ethnic groups revealed that the prevalence of overweight and obesity in PCOS women ranged from 40 to 100% [5]. Furthermore, a retrospective study suggested that embryo implantation and live birth rates were significantly lower in overweight/obese women patients than in normal-weight women with PCOS, while the late abortion rate was significantly higher [17]. Previously, few retrospective analyses evaluated the pregnancy outcomes of overweight/obese women with PCOS after treatment using different endometrial preparation protocols. This study compares the efficacy of mildly stimulated cycle (mSTC) versus AC for endometrial preparation before FET in a retrospective study of PCOS overweight/obese patients.

## Materials and methods

### Study design and participants

**Fig. 1:**
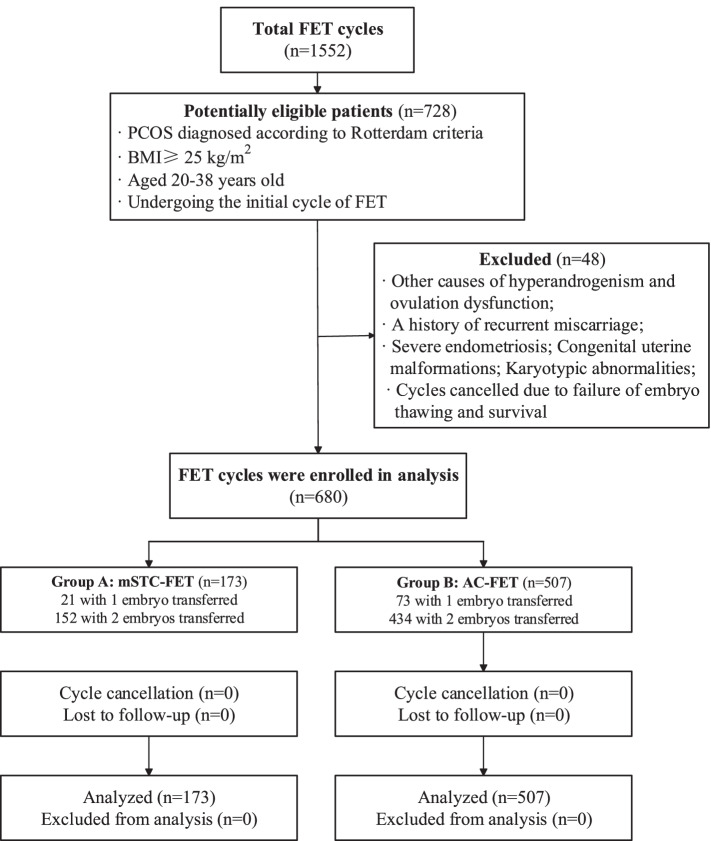
Workflow of study enrollment

This retrospective study was performed at the Reproduction and Genetics Center of Integrated Traditional Chinese and Western Medicine at the Affiliated Hospital of Shandong University of Traditional Chinese Medicine. It was approved by the Reproductive Ethics Committees of the Center (ref approval no. SDTCM20201008). Because this was a retrospective investigation, patients were not asked to participate in the analysis. This study enrolled 680 cases of PCOS overweight/obese women undergoing IVF/ICSI (intracytoplasmic sperm injection) and their first FET cycles from January 2018 to December 2020. According to Rotterdam consensus [18], PCOS was diagnosed as fulfilling at least two of the three following items: 1) oligo-anovulation or anovulation; 2) clinical or biochemical signs of hyperandrogenism; and 3) polycystic ovarian morphology on ultrasound, as defined by at least one ovary with a volume ≥ 10 cm^3^ or ≥ 12 follicles. Further inclusion criteria included participants aged between 20 and 38 years and body mass index (BMI) ≥ 25 kg/m^2^. Exclusion criteria included the following: 1) other causes of hyperandrogenism and ovulation dysfunction; 2) A history of recurrent miscarriage; 3) severe endometriosis; 4) congenital uterine malformations; 5) karyotypic abnormalities; and 6) cycles canceled due to failure of embryo thawing and survival (Fig. 1).

### Ovarian stimulation and laboratory protocols

All patients underwent one of the following two COH regimens: a flexible gonadotropin-releasing hormone antagonist (GnRH-ant) protocol or a standard GnRH agonist (GnRH-a) long protocol. Briefly, patients in the flexible GnRH-ant protocol were injected daily with 150–225 IU recombinant FSH (Gonal-F, Merck-Serono, Lyon, France) from day 2 or 3 of the menstrual cycle, with daily 0.25 mg GnRH-ant (Cetrorelix, Merck Serono, Darmstadt, Germany) being initiated once the largest follicle was > 12–14 mm in size. For the standard GnRH-a long protocol, patients underwent pituitary down-regulation using GnRH-a (Triptorelin, Decapeptyl, Ipsen, France) during the luteal phase of the previous menstrual cycle, followed by an initial dose of 150 ~ 225 U recombinant FSH for ovulation induction. Additionally, gonadotropin doses were adjusted according to the ovarian response, as monitored via ultrasonography and measurement of serum sex steroids. When ≥ 2 follicles measured ≥ 18 mm, 4000–10,000 IU human chorionic gonadotropin (hCG; Lizhu, Zhuhai, China) or 250 μg of recombinant human choriogonadotro-pinalfa solution (Ovitrelle®, 250 μg, Merck) was administered to trigger final oocyte maturation. Oocyte retrieval was performed 34–36 h later. A conventional IVF/ICSI approach was employed to fertilize the harvested oocytes in light of the specifics of a given semen sample. According to clinical practice indication, IVF/ICSI procedures were either followed by fresh embryo transfer or a freeze-all strategy.

High-quality embryos that were not used for fresh embryo transfer were cryoprotected in a closed vitrification system. For cleavage-stage embryos (day 3), the quality was graded using Cummins criteria [19], and the best quality embryos (grade I-II) were selected for vitrification. Suboptimal day-3 embryos (grade III-IV) were placed in extended culture to the blastocyst stage. Day-5 embryo quality was evaluated based on the scoring system of Gardner and Schoolcraft [20], with embryos graded ≥ 3BB classified as high-quality blastocysts. All FET cycles include the transfer of up to two embryos. On the day of transfer, all embryos were thawed, and any embryo with more than 50% blastomeres was considered transplanted. The vitrification and thawing procedures were performed as in previous reports [21].

### Endometrium preparation protocols

#### Mildly stimulated cycles

In the mildly ovarian stimulation protocol, letrozole (LE; Jiangsu Hengrui Medicine Co., China) was administered orally for five consecutive days at a daily dose of 5 mg, initiating on day 3 of a spontaneous menstrual cycle or progesterone-induced withdrawal bleeding. After that, a dosage of human menopausal gonadotropin (HMG; Anhui Fengyuan Pharmaceutical Co., China) 37.5 ~ 75 IU was administered to stimulate follicle growth. Then, 37.5 ~ 75 IU of HMG was added as appropriate according to the patient's BMI and ovarian response to stimulation. Ultrasound monitoring and serum hormone analysis were performed from cycle day 10 onwards. Transvaginal ultrasound monitoring was performed every three days. Whenever the endometrial thickness was ≥ 7 mm, and dominant follicle ≥ 17 mm, with E_2_ levels preferably > 150 pg/mL, HCG 4000 IU were injected for the final oocyte triggering. An injection of progesterone (Zhejiang Xianju Pharmaceutical Co., China), 40 mg per day, was provided two days following HCG administration. Three or five days after progesterone administration, synchronized warm embryos are transferred. After 14 days of ovarian stimulation, if the endometrial thickness did not reach 7 mm, the cycle was canceled regardless of the dominant follicle size.

#### Artificial cycles

In the artificial cycle, endometrial preparation includes sequential administration of estradiol valerate tablets (DELPHARM Lille S.A.S, France) and progesterone. On days 2–3 of the spontaneous or discontinued progesterone menstrual cycle, if endometrial thickness was less than 5 mm and serum estradiol level was less than 50 pg/mL assisted by vaginal ultrasound and blood monitoring, endometrial preparation was started by prescribing 4 mg estradiol valerate daily for five days, followed by 6 mg. On the 13^th^ day of estradiol administration, an ultrasound scan was conducted to measure endometrial thickness. If endometrial thickness was ≥ 7 mm, 40 mg daily progesterone injection was initiated. If endometrial thickness was less than 7 mm, estradiol valerate tablets were increased to 8 mg/day orally until the intima reached the appropriate thickness. Three or five days after progesterone administration, synchronized warm embryos were transferred. If the endometrial thickness remains inadequate, the cycle would be canceled. If a pregnancy is confirmed, luteal support cannot be withdrawn in mSTC and AC groups until ten weeks of gestation.

The choice of endometrial preparation depends on patient and physician preference. When physicians choose endometrial preparation for patients depending on many aspects, one of the most important aspect is curative effect. Furthermore, the cost and convenience of treatment should also be taken into account. Compared to stimulated cycle, artificial cycle has administration conveniently, good operability and strong time flexibility. Therefore, artificial cycle is often used as the first choice for FET endometrial preparation in patients with PCOS. The mildly stimulated cycles is usually used in the following situations: multiple FET failures in artificial cycles, poor response to exogenous estrogen in previous artificial cycles and E2 < 100 pg/ml during hormone replacement cycle. However, frozen embryo transfer has been a standardized treatment for PCOS patients since 2012 at our reproduction center.

### Outcome measures

The primary outcome of the study was live birth rate (LBR), and the secondary outcomes included implantation rate, positive pregnancy rate, clinical pregnancy rate, endometrial thickness, ectopic pregnancy rate, and pregnancy loss rate. Implantation rates were determined via ultrasound-mediated assessment of how many gestational sacs were evident in a patient compared to the number of transferred embryos. Positive pregnancy was defined as a serum β-hCG level > 10 IU/L at 14 days after cleavage embryo transfer. If β-hCG assay yielded a positive result, clinical pregnancy was established as the presence of at least one gestational sac in the uterine cavity on ultrasound five weeks after FET. Live birth was defined as a live-born baby after ≥ 24 gestational weeks. The pregnancy loss was defined as a loss of clinical pregnancy within 24 weeks. Ectopic pregnancy refers to abnormal pregnancy in which fertilized egg implants outside the uterine cavity and can be diagnosed by ultrasound, surgical visualization, or histopathology.

### Statistical analysis

Statistical analysis was performed using Statistical Package for Social Sciences (SPSS) version 21.0 (SPSS Inc., Chicago, USA). The continuous variables were expressed as mean ± standard deviations (SD) and were compared between groups using Student’s *t*-test. The Chi-square (*χ*^*2*^) statistics was conducted to compare categorical variables between two groups, and the results were expressed as numbers/percentages. Multivariate logistic regression analysis was utilized to examine the possible effects of the following known potential confounding factors on pregnancy outcomes of FET cycle, including age at FET, BMI, infertility duration, infertility type (primary or secondary), ovulation induction regimen (agonist regimen or antagonist regimen), gonadotropin (Gn) usage time, Gn dosage, oocytes retrieved, laboratory fertilization mode (IVF or ICSI), number of embryos transferred, embryo stage and quality at transfer, and endometrium thickness prior to FET. A *P*-value < 0.05 was considered statistically significant.

## Results

### Baseline and ART characteristics of patients

The current study analyzed 680 FET cycles. Specifically, 173 (25%) patients received mSTC, and 507 (75%) patients underwent AC before FET. Patients’ baseline characteristics are detailed in Table 1. No significant difference was observed between the two treatment groups regarding age, infertility duration, BMI, or type of infertility (*P* > 0.05). The basal serum FSH and LH levels was similar between the two groups (*P* > 0.05). Moreover, there was no significant difference in the days of ovarian stimulation and total dose of gonadotrophin (Gn) between the two patient groups (*P* > 0.05). The ovulation induction regimens used were agonist (496 cases, 72.9%), antagonist (184 cases, 27.1%), laboratory IVF (573 cases, 85.7%), and ICSI (107 cases, 14.3%). In mean values, 22 oocytes were retrieved, and 7 embryos were frozen.

**Table 1:** Baseline characteristics of ovarian stimulation and IVF cycles

	Total	mSTC	AC	*P* value
Number of patients	680	173	507	
Age of woman (years)	30.10 ± 3.22	30.31 ± 3.49	30.20 ± 3.12	0.346
Infertility duration(years)	3.25 ± 2.03	3.05 ± 1.97	3.32 ± 2.05	0.122
BMI (kg/m^2^)	27.77 ± 4.33	27.40 ± 4.19	27.90 ± 4.37	0.122
Baseline hormonal profile				
FSH (mIU/mL)	6.40 ± 1.56	6.39 ± 1.67	6.40 ± 1.52	0.982
LH (mIU/mL)	8.32 ± 4.60	8.58 ± 5.01	8.22 ± 4.45	0.374
Type of infertility				0.514
Primary infertility	380(55.9%)	93(53.8%)	287(56.6%)	
Secondary infertility	300(54.1%)	80(46.2%)	220(43.4%)	
Laboratory insemination				0.162
IVF	573(85.7%)	140(80.9%)	433(87.4%)	
ICSI	107(14.3%)	33(19.1%)	74(12.6%)	
Ovulation induction regimen				0.406
Agonist regimen	496(72.9%)	122(70.5%)	374(73.8%)	
Antagonist regimen	184(27.1%)	51(29.5%)	133(26.2%)	
Gn days (days)	11.36 ± 2.96	11.58 ± 3.06	11.29 ± 2.92	0.262
Total Gn (mIU/mL)	2287.28 ± 1001.74	2388.54 ± 1031.18	2252.73 ± 990.16	0.124
Number of eggs obtained	22.26 ± 9.48	24.40 ± 4.19	22.13 ± 9.614	0.189
Total number of frozen embryos	6.94 ± 2.36	7.18 ± 2.43	6.85 ± 2.33	0.107

Data are presented as mean ± SD for continuous variables and n (%) for dichotomous variables. All* P* values were assessed with the use of student’s t-test or *χ*^*2*^

*BMI* Body Mass Index, *FSH* Follicle Stimulating Hormone, *Gn* Gonadotropin, *ICSI* Intracytoplasmic Sperm Injection, *IVF* In Vitro Fertilization, *LH* Luteinizing Hormone

### Cycle characteristics of FET

As presented in Table 2, no significant differences were found regarding number of embryos transferred, embryo grades and stages between two groups (*P* > 0.05). Most patients were transferred double embryos (86.2%) of D3 stage (87.8%). However, the mSTC group exhibits significantly greater endometrial thickness prior to FET (10.10 ± 1.94 vs. 9.75 ± 1.61, *P* < *0.05*).

**Table 2:** FET cycle characteristics between groups

	Total	mSTC	AC	*P* value
Number of patients	680	173	507	
Number of embryos transferred				0.457
Single	94(13.8%)	21(12.1%)	73(14.4%)	
Double	586(86.2%)	152(87.9%)	434(85.6%)	
Embryo grades				0.382
Level 1	446(35.2%)	108(33.2%)	338(36.0%)	
Level 2	820(64.8%)	217(66.8%)	603(64.0%)	
Embryos stages				0.402
Day 3 embryos	597(87.8%)	155(89.6%)	442(87.2%)	
Day 5 embryos	83(12.2%)	18(10.4%)	65(12.8%)	
Endometrium thickness prior to FET (mm)	9.84 ± 1.71	10.10 ± 1.94	9.75 ± 1.61	**0.039**

Data are presented as mean ± SD for continuous variables and n (%) for dichotomous variables. All* P* values were assessed with the use of student’s t-test or *χ*^*2*^

### Pregnancy outcomes

Our findings of pregnancy outcomes in these two different FET groups are described in Table 3. The LBR were 49.7% and 41.0% following mSTC-FET and AC-FET, respectively, reaching statistical significance (*P* < *0.05*). The rate of singletons and twins among the live-birth deliveries between the two groups had no significant difference (*P* > 0.05). Compared with AC group, those in the mSTC group exhibited a significantly lower miscarriage rate (6.4% vs. 23.0%; *P* < *0.001*). However, no statistical significance was observed between two groups in implantation rates (mSTC: 34.2% vs. AC: 36.9%; *P* > 0.05), positive pregnancy (mSTC: 57.8% vs. AC: 60.0%; *P* > 0.05), and clinical pregnancy (mSTC: 54.3% vs. AC: 55.6%; *P* > 0.05). Moreover, the rates of ectopic pregnancy (mSTC: 2.1% vs. AC: 3.2%; *P* > 0.05) and twin pregnancies (mSTC: 20.2% vs. AC: 24.8%; *P* > 0.05) were similar between two groups.

**Table 3:** Pregnancy outcomes between groups

	Total	mSTC	AC	*P* value
Number of patients	680	173	507	
Implantation rate	458/1266 (36.2%)	111/325(34.2%)	347/941(36.9%)	0.379
Positive pregnancy rate	404/680 (59.4%)	100/173(57.8%)	304/507(60.0%)	0.618
Clinical pregnancy rate	376/680 (55.3%)	94/173(54.3%)	282/507(55.6%)	0.769
Twin pregnancies	69/376 (18.4%)	19/94(20.2%)	70/282(24.8%)	0.362
Ectopic pregnancy	11/376 (3.0%)	2/94(2.1%)	9/282(3.2%)	0.860
Miscarriage rate	68/376 (18.1%)	6/94(6.4%)	65/282(23.0%)	**< *0.001***
Live birth rate	294/680 (43.2%)	86/173(49.7%)	208/507(41.0%)	***0.046***
Singletons	235/680 (34.6%)	70/173(40.5%)	165/507(32.5%)	0.059
Twins	59/680 (8.7%)	16/173(9.2%)	43/507(8.5%)	0.757

Data are presented as mean ± SD for continuous variables and n (%) for dichotomous variables. All* P* values were assessed with the use of student’s t-test or *χ*^*2*^

After adjusting for the confounding factors between endometrial preparation regimes and pregnancy outcomes (Table 4), LBR remained consistently higher following mSTC group (adjusted odds ratio [aOR] 1.462, 95% confidence interval [CI] 1.028–2.079). And the miscarriage rate in the mSTC group was still significantly lower than in the AC group (aOR 0.237, 95% CI 0.100–0.562). Furthermore, in the crude and adjusted models, the mSTC group was comparable to the AC group in terms of positive and clinical pregnancy rates.

**Table 4:** Binary logistics regression analysis with pregnancy outcomes as the influencing factor

Pregnancy Outcomes	Endometrial Preparation	Crude Model		Adjusted Model	
		OR(95% CI)	*P* value	OR(95% CI)	*P* value
Positive pregnancy rate	mSTC	0.915(0.645–1.298)	0.618	0.920(0.646–1.312)	0.646
	AC				
Clinical pregnancy rate	mSTC	0.949(0.671–1.343)	0.769	0.950(0.669–1.351)	0.777
	AC				
Miscarriage rate	mSTC	0.244(0.104–0.574)	** < *0.001***	0.237(0.100–0.562)	** < *0.001***
	AC				
Live birth rate	mSTC	1.393(0.986–1.970)	***0.046***	1.462(1.028–2.079)	***0.035***
	AC				

Analyses were adjusted for age at FET, body mass index (BMI), infertility duration, infertility type (primary, secondary), ovulation induction regimen (Agonist regimen, Antagonist regimen), Gn usage time, Gn dosage, oocytes retrieved, laboratory fertilization mode (IVF, ICSI), number of embryos transferred, embryo stage and quality at transfer, endometrium thickness prior to FET,

## Discussion

Obesity and PCOS are closely related disorders with overlapping features, which may negatively impact pregnancy or neonatal outcomes [22]. So far, few studies have evaluated different preparation methods of endometrium before FET in overweight/obese women with PCOS. In this retrospective study of 680 overweight/obese women with PCOS undergoing FET, pregnancy outcomes of 173 stimulated cycles were retrospectively evaluated with reference to outcomes of 507 artificial cycles. We provided clinically relevant evidence suggesting that, compared with AC-FET group, the mSTC-FET group had significantly higher LBR and a significantly lower rate of pregnancy losses.

The abnormalities of basal hormone levels in PCOS patients have presented as high levels of androgens and LH, as well as low levels of FSH and E_2_, which might have unpleasant effects on the development of follicles, ovulation, and endometrial receptivity, eventually leading to infertility. Potential mechanisms of obesity-related PCOS included insulin resistance and hyperinsulinemia, lipotoxicity secreted by excessive free fatty acids, adipokines produced by abnormal secretion of adipocytes, and inflammatory reaction induced by obesity [23]. Numerous factors influence the pregnancy outcomes of FET, such as the age of patients, the quality of embryos, endometrial receptivity, and so on, among which endometrial receptivity is one of the key factors. So far, there is no comparative study identifying the best protocol to prepare the endometrium in overweight/obese women with PCOS before FET.

A large retrospective study of 1556 women with PCOS undergoing FET found that overweight and obese women achieved similar pregnancy outcomes, including implantation rates, clinical pregnancy, and live birth, as did normal-weight women when the quality of thawed embryos transferred was similar. It was concluded that obesity has a detrimental effect on embryo quality while not affecting endometrial receptivity or early implantation [22]. Zhang et al.’s large retrospective study confirmed our findings that letrozole-stimulated cycles (L-FET) had significantly higher LBR and lower pregnancy loss rate than AC-FET [24]. But their findings are subject to the biases of the statistical models used. A significantly higher LBR was found in patients treated with letrozole only in an adjusted analysis, which need to be taken into consideration when interpreting their results. Another retrospective cohort study conducted in Japan by Tatsumi et al. indicated that letrozole stimulation during FET resulted in higher clinical pregnancy and LBR and a lower risk of miscarriage than substituted and natural cycles [25]. Their results were supported by an exceedingly large sample size, with a total of 110,722 patients included (2409 in letrozole group, 66,843 in the AC group and 41,470 in natural group). But this study lacked information concerning the reasons for selecting the specific FET method, parity, the number of previous ART failures, embryo quality and the dose and duration of letrozole intake. In addition, the authors mentioned that in their country, letrozole was primary prescribed to patients with unexplained infertility rather than PCOS patients. To corroborate the findings of Tatsumi et al., a more recent retrospective cohort study conducted in Israel concluded that ovulation induction using letrozole might induce normal ovulation and physiological FET, resulting in better pregnancy rates in FET treatments for PCOS patients [26]. A recent historical cohort analysis on women with PCOS found that ovarian stimulation (OS) protocol with low doses of HMG achieved a higher LBR than hormone replacement therapy (HRT) protocol accompanied by the poorest endometrial thickness [27]. This cohort study was the first and the largest to compare natural, OS and HRT cycles for endometrial preparation in women with PCOS. However, as a historical analysis, bias could have been introduced because the women in natural group had a lower BMI and lower LH and total testosterone levels. Although Peigne et al. concluded similar clinical pregnancy rates (24.4% vs. 20.8%) between AC-FET and mSTC-FET groups, LBR (17.1% vs. 9.8%) was significantly higher with mSTC than with AC group, even after adjusting for potential bias [28]. But this study included all 1021 autologous FET, among them, PCOS patients accounted for 18%. In contrast with our findings, Azadeh et al., in a randomized clinical trial, observed no difference in pregnancy outcomes and cancellation rates between the letrozole plus HMG method and the artificial FET protocol [29]. Noting that this study was the largest randomized clinical trial design to compare the effect of mSTC with AC on pregnancy outcome in patients with PCOS; yet, this trial could not find a significant effect to prioritize the use of mildly stimulated cycle for endometrial preparation over the hormonal method, which may be due to sample size limitation. In Yu et al.’s retrospective study, endometrial thickness was similar in AC-FET and STC-FET. The two protocols resulted in non-statistically different rates of clinical pregnancy, ongoing pregnancy, live birth. Nevertheless, the STC-FET had a significantly higher cancelled cycle rate [30]. However, the present study was a study with a small size of the sample. Besides, two recent meta-analyses comparing L-FET with AC-FET group in PCOS patients found no difference between two groups for LBR, whereas a lower miscarriage rate was found for the L-FET than AC-FET [31, 32].

The ovarian stimulation protocol imitates the natural process of follicular development using ovulation stimulants to assist endogenous estradiol synthesis, thereby promoting endometrial growth. In this study, ovulation stimulants were letrozole combined with low doses of HMG. Letrozole is a third-generation aromatase inhibitor drug that, when used in the early stage of promoting ovulation, can decrease intraovarian and serum estrogen levels by blocking the conversion of androgens to estrogens in the ovarian granulosa cells without antagonistic effects on estrogen receptors. However, its estrogen-lowering effect is significantly reduced in the late follicular stage [33]. This process led to a rapid thickening of endometrium and increased blood level in the uterus and endometrium, positively impacting pregnancy outcomes [34]. Our study revealed that the letrozole group demonstrated significantly greater endometrial thickness than the artificial cycle group prior to FET. A previous study has indicated that using letrozole for ovulation induction focused on PCOS patients improved endometrial receptivity, which might positively affect embryo implantation [35]. Likewise, another preliminary study observed that ovarian stimulation using letrozole resulted in a sevenfold increase in the expression of uterine receptivity markers, including integrin, leukemia inhibitory factor, and L-selectin, in women with unexplained infertility compared with spontaneous cycles [36], all of which may correlate with a higher success rate of embryo transfer. Moreover, a recent population-based study conducted in Japan indicated that letrozole administration during IVF cycles neither increased the risk of major congenital anomalies nor compromised neonatal outcomes of IVF newborns compared with natural cycles, indicating that letrozole is relatively safe [37]. Previously, studies demonstrated that the lack of a corpus luteum would perturb maternal circulation, increasing preeclampsia incidence. Human menopausal gonadotropin (HMG), which contains follicle stimulating hormone and luteinizing hormone, can secrete gonadotropin to promote follicle maturation, so as to stimulate ovulation and to accelerate the development of corpus luteum. Chen et al. found that letrozole in combination with low-dose intramuscular injection of HMG had satisfactory therapeutic effects on ovulation induction, short medication cycle and high clinical pregnancy rate [38]. Another study showed that using letrozole alone to endometrial preparation programs for FET led to a lower estrogen level or a thinner endometrium when the follicle matured. Which led to a lower pregnancy rate and a higher cycle cancellation rate after FET. Letrozole combined with a small doses of HMG could promote the synthesis of estrogen and improve the endometrium [39]. Mild ovarian stimulation with low doses of gonadotropins could induce the development of a proper number of follicles and ovulation, thereby ensuring better function of corpus luteum [40, 41]. And it is best to take letrozole orally and then inject HMG intramuscularly to achieve the purpose of single follicle development [42]. However, because women with PCOS are more likely to develop OHSS than those without PCOS, women undergoing ovarian stimulation should receive a low initial dose of gonadotropins and be monitored frequently to ensure that only one or two dominant follicles are allowed to grow during endometrial preparation. Once exposed to OHSS risk or no dominant follicle, the transplant cycle would be canceled. Financial affordability is a major issue for patients seeking infertility treatment. In China, letrozole plus HMG is relatively inexpensive. Nonetheless, the ovarian stimulation protocol requires more monitoring via ultrasound examinations and endocrine, resulting in increased expenses. However, this protocol is relatively safe and is associated with increased pregnancy rates. Additional research examining the cost-effectiveness of these two regimens is required.

The artificial hormone replacement endometrial preparation protocol utilizes exogenous estradiol to simulate endocrine environment and promote endometrial development consistent with that observed in a normal menstrual cycle [43]. Although few studies demonstrate that hormone replacement therapy results in relatively unsatisfactory pregnancy outcomes, those who accept the subject point out that adverse outcomes of hormone replacement therapy cycle could be due to excessive intake of estradiol. Ma et al. found that uterine receptivity window remains open for a long time at low estrogen level but closes rapidly at high estrogen level [44]. Moreover, while estrogen is applied at the early stage of follicles in the hormone replacement cycle, sometimes it cannot completely inhibit their development, and developing follicles can still secrete steroid hormones. The combined action of endogenous and exogenous steroid hormones may lead to imbalanced estrogen-progesterone proportion in the implant window, hence decreasing receptivity [45]. In addition, a deficient luteal phase in the AC-FET group could be the cause of the heightened risk for suffering miscarriages with an asynchronized window of implantation. An optimal progesterone level seems to be necessary in AC-FET group for the evolution of pregnancy. Kofinas et al. indicated that a level of progesterone over 20 ng/ml on day of blastocyst transfer was associated with a increased miscarriage rate [46]. A study on the optimal duration of progesterone administration before FET with an AC-FET confirmed a higher early miscarriage rate when the period of progesterone supplementation was too short [47]. Adaptation of AC-FET treatment according to progesterone levels was suggested as a way to improve ongoing pregnancy rates and decrease pregnancy losses [28]. In mSTC group, a corpus luteum is active, secreting progesterone progressively until stabilization, leading to a more constant progesterone level [48]. Moreover, the final oocyte triggering with HCG could provide a luteotropic effect in the early luteal phase [30]. Finally, other factors are also secreted by the corpus luteum, leading to different (possibly more natural) endometrial protein secretion profiles than with an AC-FET [48]. Yu et al. suggest that PCOS women and chronic anovulation may be less responsive to exogenous estradiol [30], consistent with our study that artificial hormone replacement group had a lower LBR and poorer endometrial thickness before embryo transfer. These studies may provide evidence for low LBR and high miscarriage rate in HRT group. However, the main disadvantage of this method is the adverse effects of employed hormones, such as the risk of maternal thromboembolic events and genital malformations in male fetuses [29]. Moreover, once pregnancy is confirmed, exogenous estrogen and progesterone should be used until the placenta is formed to replace the absent corpus luteum. Most importantly, exogenous hormones may be inadequate in some patients for proper endometrial development.

A major weakness of this study is its retrospective format, and the potential heterogeneity in patient characteristics such as the top quality embryos have already been transferred in the fresh embryo transfer cycle that makes the population less comparable. Although we adjusted our analyses to minimize the likelihood of confounding, it is impossible to completely preclude the possibility of underlying selection bias. In addition, some physicians prefer the artificial hormone replacement protocol over the ovarian stimulation protocol because it avoids inducing OHSS using gonadotropins. Although the dosage of ovulation induction drugs used in the stimulated protocol is safe, a large sample size difference was observed between the two groups. However, this study also has some strengths, including a larger cohort size with a recent 100% follow-up. In addition, our study was carried out in a single center. Thus, it will be interesting to validate these findings in a multi-center clinical trial over a shorter period of time in the future. Moreover, our study will be more complete and persuasiveness if obstetric and neonates outcomes are followed-up.

## Conclusion

In conclusion, the mSTC-FET demonstrated a higher LBR and lowered pregnancy loss rates than the AC-FET in overweight/obese women with PCOS. This study, coupled with existing evidence, confirmed that mildly stimulated cycles result in superior pregnancy outcomes than artificial cycles. These findings imply that using letrozole plus HMG for endometrial preparation might be a potentially better alternative for overweight/obese women with PCOS when considering the most cost-effective treatment with the least adverse effects on patients. To corroborate our findings, prospective randomized trials are necessary to determine the efficacy of mildly stimulated cycles as a method of endometrial preparation for FET.

## Supplementary Information

**Additional file 1.** 

## Data Availability

The data that supports the findings of this study are available in the supplementary material of this article.

## Abbreviations

AC: Artificial cycles
ART: Assisted reproductive techniques
BMI: Body mass index
COH: Controlled ovarian hyperstimulation
FET: Frozen embryo transfer
HCG: Human chorionic gonadotropin
HMG: Human menopausal gonadotropin
HRT: Hormone replacement therapy
ICSI: Intracytoplasmic sperm injection
IVF: In vitro fertilization
LBR: Live birth rate
mSTC: Mildly stimulated cycles
OHSS: Ovarian hyperstimulation syndrome
OS: Ovarian stimulation
PCOS: Polycystic ovary syndrome
